# Crosstalk between autophagy and microbiota in cancer progression

**DOI:** 10.1186/s12943-021-01461-0

**Published:** 2021-12-11

**Authors:** Yu Wang, Jiang Du, Xuemei Wu, Ahmed Abdelrehem, Yu Ren, Chao Liu, Xuan Zhou, Sinan Wang

**Affiliations:** 1 Department of Maxillofacial and Otorhinolaryngological Oncology, Tianjin Medical University Cancer Institute and Hospital, Tianjin, 300060 China; 2 Key Laboratory of Cancer Prevention and Therapy, Tianjin Cancer Institute, Tianjin, 300060 China; 3 National Clinical Research Center of Cancer, Tianjin, 300060 China; 4 Department of Gastroenterology and Hepatology, Tianjin Medical University General Hospital, Tianjin, China; 5 Tianjin Gastroenterology and Hepatology Institute, Tianjin Medical University, Tianjin, 300052 China; 6 Key Laboratory of Immune Microenvironment and Disease, Tianjin Medical University, Ministry of Education, Tianjin, 300070 China; 7 Department of Craniomaxillofacial and Plastic Surgery, Faculty of Dentistry, Alexandria University, Alexandria, Egypt; 8 Tianjin Research Center of Basic Medical Science, Tianjin Medical University, Tianjin, 300070 China

**Keywords:** Autophagy, Microbiota, Cancer progression, Target therapy

## Abstract

Autophagy is a highly conserved catabolic process seen in eukaryotes and is essentially a lysosome-dependent protein degradation pathway. The dysregulation of autophagy is often associated with the pathogenesis of numerous types of cancers, and can not only promote the survival of cancer but also trigger the tumor cell death. During cancer development, the microbial community might predispose cells to tumorigenesis by promoting mucosal inflammation, causing systemic disorders, and may also regulate the immune response to cancer. The complex relationship between autophagy and microorganisms can protect the body by activating the immune system. In addition, autophagy and microorganisms can crosstalk with each other in multifaceted ways to influence various physiological and pathological responses involved in cancer progression. Various molecular mechanisms, correlating the microbiota disorders and autophagy activation, control the outcomes of protumor or antitumor responses, which depend on the cancer type, tumor microenvironment and disease stage. In this review, we mainly emphasize the leading role of autophagy during the interaction between pathogenic microorganisms and human cancers and investigate the various molecular mechanisms by which autophagy modulates such complicated biological processes. Moreover, we also highlight the possibility of curing cancers with multiple molecular agents targeting the microbiota/autophagy axis. Finally, we summarize the emerging clinical trials investigating the therapeutic potential of targeting either autophagy or microbiota as anticancer strategies, although the crosstalk between them has not been explored thoroughly.

## Background

Autophagy is an evolutionarily conserved intracellular recycling and cellular self-degradation process that occurs in eukaryotes and plays a critical role in the maintenance of homeostasis in various biological processes [1, 2]. Many studies have indicated that autophagy, as a cell death mechanism, plays an important pathophysiological role in various disease processes, including cell death, infection, heart diseases, neurodegeneration, autoimmune diseases and cancer [3–8]. Autophagy serves multiple functions in cancer progression by modulating cell death, and further studies have shown that autophagy plays a dual role in cancer. It can promote malignant transformation in certain tumors and suppress tumor growth in others [1, 2, 9]. Previous reports showed that BECN1, an essential autophagy-related gene (ATG), was deleted in 40 to 75% of breast, ovarian, and prostate cancers, suggesting the role of autophagy in tumor growth suppression [10–12]. In addition, autophagy is upregulated in RAS-transformed tumors, hence promoting their survival, growth and tumorigenesis [13–15]. Autophagy can be classified based on the mechanism into common (nonselective) or selective types. Common autophagy involves the packaging of cytoplasmic portions into autophagosomes and the delivery of these cargoes to lysosomes for degradation. In contrast, selective autophagy is activated when specific targets, such as protein aggregates, damaged cell organelles, and intracellular pathogens, are recognized [16–18].

The correlation between cancer and microbiota is still unclear. It is well known that genetic and environmental factors are critical for the initiation and progression of cancer, but recent studies have shown that microorganisms are also indispensable [19]. Gastric [20], ovarian [21], pancreatic [22], prostate [23], lung [24] and breast cancers [25], in addition to cholangiocarcinoma [26], have all been found associated with microorganism infection. Recently, mucosa-associated microbiota was shown to be essential for the microenvironment of various malignant tumors; moreover, intratumoral organisms can influence tumorigenesis and metastasis [27–31]. Garrett W.S. also indicated that the ways in which microbes and microbiota contribute to carcinogenesis, whether by enhancing or diminishing a host’s risk, fall into three broad categories: (i) altering the balance of host cell proliferation and death, (ii) guiding immune system function, and (iii) influencing the metabolism of host-produced factors, ingested foodstuffs, and pharmaceuticals [32]. Thus, micromolecular drugs targeting microorganisms have become a research hotspot in the context of antitumor treatment, mainly for tumors caused by microbial infections. Moreover, certain microorganisms that can affect the response of tumors to other therapy strategies can be used to treat cancer, referred as “use microorganisms to treat microorganisms” [33, 34].

Aside from their respective functions in pathological and physiological conditions, the crosstalk between autophagy and microbiota is vital in tumorigenesis and resistance to chemotherapy drugs [35, 36]. Autophagy induced by *Porphyromonas gingivalis* infection controls cell proliferation and G1 arrest in host oral cancer cells [35]. *Fusobacterium nucleatum* promotes cancer by upregulating ULK1 and ATG7 to induce resistance to oxaliplatin and 5-fluorouracil (5-FU) in colorectal cancer (CRC) [36]. More studies on the crosstalk between microbiota and autophagy are needed to better explore different variables and define the correlation, as well as the mechanisms through which they affect cancer progression. A deeper understanding of the molecular mechanisms of the microbiota/autophagy axis may help to design improved anticancer drugs for clinical use.

In this review, we described the critical roles of microbiota and autophagy in tumors separately and their characteristics in tumor progression. Moreover, this review analyzed the effects and mechanisms of autophagy regulated by different bacteria on the biological behavior of tumors, as well as the impact of autophagy on the carcinogenesis of microbiota, emphasizing the dominant role of autophagy in the interaction between the microbiota and human tumors. In addition, multiple drugs were summarized, and clinical trials or animal experiments were evaluated to assess the therapeutic potential of targeting the microbiota/autophagy axis as anticancer strategies.

## Overview of microbiota and autophagy

### Microbiota and cancer

**Fig. 1:**
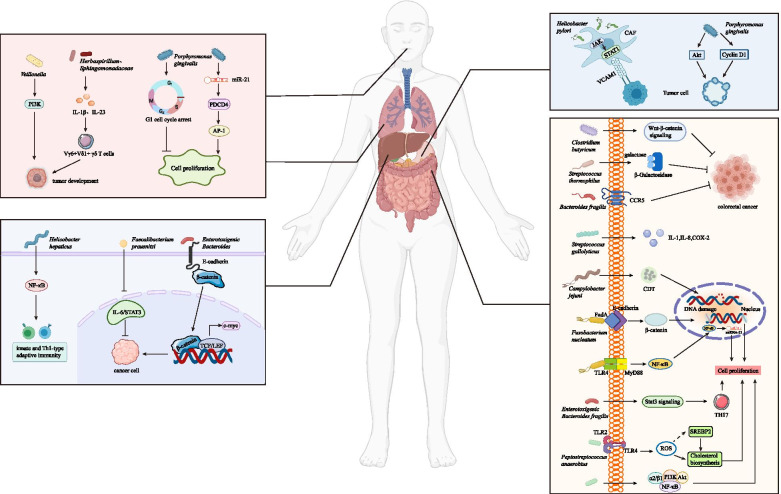
Overview of the mechanisms by which bacteria regulate tumor progression. Pathogenic bacteria mainly come from oral and gastrointestinal flora. Different kinds of bacteria can promote the occurrence, development and metastasis of tumors by causing host adaptive immune responses, cell cycle arrest, DNA transcription changes and DNA damage, reactive oxygen species accumulation and the activation of various signaling pathways, such as Wnt/β-catenin, NF-κB, and STAT3 signaling

During cancer development, the microbial community may have an indirect carcinogenic function by promoting mucosal inflammation or causing systemic disorders [37], where most studies focused on the oral and intestinal microbiota (Fig. 1).

The oral microbiome is very complex, and common pathogenic bacteria include *Streptococcus anginosus, Veillonella, F. nucleatum* and *P. gingivalis* [38, 39]. Studies have shown that pathogenic bacteria in the oral cavity are associated with multiple cancers of the digestive system [38]. The development of esophageal carcinoma has been reported to be related to *P. gingivalis* [40] and *F. nucleatum* [41]. The detection rate of *P. gingivalis* in esophageal squamous cell carcinoma (ESCC) is higher than that in adjacent tissue or normal controls [42]. In addition, *P. gingivalis* might utilize the miR-194/GRHL3/PTEN/AKT axis to promote ESCC proliferation and migration [43]. The *F. nucleatum* DNA concentration in esophageal cancer tissue is significantly higher than that in normal esophageal tissue [41]. The levels of oral bacteria (such as *Streptococcus* and *Clostridium*) in gastric cancer tissue are significantly higher than those in normal tissue, while *Lactobacillus brevis* is less enriched than that in the non-tumor tissue conversely [ 44]. The proportions of *Bacteroides* and *Firmicutes* in patients with non-cardia gastric cancer decrease significantly [ 45]. The abundance of *Neisseria elongata* and *Streptococcus mitis* in the salivary microbiome of pancreatic cancer patients are significantly lower than those in the healthy population [46]. Researchers also found a significantly higher ratio of *Leptotrichia* to *Porphyromonas* in the saliva of patients with pancreatic cancer than in that of healthy patients or those with other diseases [47]. In a controlled study, *P. gingivalis* and *F. nucleatum* were found to be associated with a higher risk of pancreatic cancer [48]. *P. gingivalis* can promote the proliferation of pancreatic cancer cells without the function of TLR2 [49]. Studies have shown that the total abundance of *F. nucleatum* in CRC is 415 times higher than that in adjacent normal tissue, and a positive correlation with lymph node metastasis has also been observed [50].

Certain bacteria in the gastrointestinal tract are associated with cell dysplasia and carcinogenic effects. *Campylobacter jejuni* [51] and *F. nucleatum* [52] have been shown to be cancer-causing intestinal bacteria in CRC. *Helicobacter pylori* increases the expression of VCAM1 in cancer-associated fibroblasts (CAFs) via JAK/STAT1 signaling pathway in gastric carcinoma, and the level of VCAM1 in patients with gastric cancer is positively correlated with tumor progression and a poor prognosis. Moreover, the interaction between CAF-derived VCAM1 and integrin αvβ1/5 could promote gastric cancer cell invasion both in vitro and in vivo [53]. In a study of CRC, it was found that chronic inflammation can be promoted via the accumulation of certain bacteria (such as *Escherichia coli*) and induce carcinogenesis by cell lethal expansion toxin produced by *C. jejuni* [51]. Furthermore, oncogenic transcriptional changes in CRC cell lines are associated with FadA adhesion complexes of *F. nucleatum* [27, 52]. *F. nucleatum* level is found increased in the stool samples of colorectal adenoma and cancer patients, and is also enriched in adenocarcinomas and adenomas compared with normal colonic tissues [54]. *F. nucleatum* increases the expression of miR-21 through the TLR4/MYD88/ NF-κB pathway and induces CRC cell proliferation and migration [55]. *Pks*^*+*^ *E. coli* could output DNA adducts and then strengthen the ability of colibactin to generate mutations in tumor suppressor genes or oncogenes, which contribute to cancer initiation and progression in mammalian and mouse cells [56]. *Enterotoxigenic Bacteroides fragilis (ETBF)*, a common commensal bacterium in human intestine, strongly induces colonic tumors in multiple intestinal neoplasia (Min) mice via STAT3- and Th17- dependent pathways [57, 58]. *Peptostreptococcus anaerobius* is more abundant in the stool samples of CRC patients than in those of normal controls without CRC [59], which could combine with α2/β1 integrin through its surface protein PCWBR2 [60]. The crosstalk between PCWBR2 and α2/β1 integrin could induce focal adhesion kinase phosphorylation and then activate the PI3K/AKT pathway in CRC cells, increase cell proliferation, activate NF-κB signaling, and finally contribute to chronic inflammation and tumor progression [60]. The abovementioned bacteria and other cancer-related microbiota are all summarized in Table 1.

**Table 1:** Microbiota involved in cancer progression

System	Cancer type	Microbiota	Tumor promotion /Tumor suppression	Tumor Behavior	Molecular Mechanism	Ref.
Digestive System	Colorectal Cancer	*Fusobacterium nucleatum*	Tumor promotion	Proliferation and invasion	*F. nucleatum* regulates E-cadherin/β-catenin signaling pathway to promote colorectal proliferation and invasion.	[52]
			Tumor promotion	Proliferation	*F. nucleatum* activates TLR4 and upregulates miR-21 to promote colorectal cancer proliferation.	[55]
		*Enterotoxigenic Bacteroides fragilis (ETBF)*	Tumor promotion	Tumorigenesis	*ETBF* selectively activates STAT3 and induces TH17 inflammatory infiltrates for enhancing tumor growth.	[57]
		*Peptostreptococcus anaerobius*	Tumor promotion	Proliferation and dysplasia	*P. anaerobius* interacts with TLR2 and TLR4 to increase intracellular ROS level, thus increases colon proliferation and dysplasia*.*	[59]
			Tumor promotion	Initiation and proliferation	*P. anaerobius* drives CRC tumorigenesis via PCWBR2/ PI3K/AKT/NF-κB signaling axis.	[60]
		*Campylobacter jejuni*	Tumor promotion	Initiation and proliferation	*C. jejuni* induces DNA damage and promotes colorectal tumorigenesis and growth via cytolethal distending toxin.	[51]
		*Streptococcus gallolyticus*	Tumor promotion	Initiation	*S. gallolyticus* promotes normal or premalignant colorectal tissues into malignant tumor via IL-1, COX-2, and IL-8 induction.	[61]
		*Clostridium butyricum*	Tumor suppression	Proliferation and metastasis	*C. butyricum* inhibits intestinal tumor development by modulating Wnt signaling and gut microbiota.	[62]
		*Bacteroides fragilis*	Tumor suppression	Cancer development	*B. fragilis* prevents colitis-associated CRC by inhibiting the expression of CCR5.	[63]
		*Streptococcus thermophilus*	Tumor suppression	Tumorigenesis	*S. thermophilus* secretes β-Galactosidase to inhibit tumorigenesis.	[64]
	Gastric Cancer	*Helicobacter pylori*	Tumor promotion	Invasion	*H. pylori* infection increase VCAM1 expression in CAFs via JAK/STAT1 signaling pathway to facilitate tumor invasion.	[53]
	Esophageal Carcinoma	*Porphyromonas gingivalis*	Tumor promotion	Proliferation and migration	*P. gingivalis* promote ESCC proliferation and migration via the miR-194/GRHL3/PTEN/ AKT signaling axis	[43]
	Pancreatic Cancer	*Porphyromonas gingivalis*	Tumor promotion	Proliferation	*P. gingivalis* enhances tumor cell proliferation through strengthening AKT signaling and Cyclin D1 expression.	[49]
	Liver Cancer	*Helicobacter hepaticus*	Tumor promotion	Proliferation	*H. hepaticus* promotes HCC by activating NF-κB regulated networks associated with innate and Th1-type adaptive immunity.	[65]
	Oral Cancer	*Porphyromonas gingivalis*	Tumor suppression	Proliferation	*P. gingivalis* inhibits proliferation of oral cancer cells by inducing G1 cell cycle arrest.	[35]
			Tumor promotion	Proliferation	*P. gingivalis* actives the miR-21/PDCD4/AP-1 signaling pathway to promote the proliferation of oral cancer.	[66]
Non-Digestive System	Lung Cancer	*Herbaspirillum*	Tumor promotion	Proliferation	*Herbaspirillum* stimulates IL-1β and IL-23 production, induces activation of Vγ6^+^Vδ1^+^ γδ T cells and tumor cell proliferation.	[67]
		*Veillonella*	Tumor promotion	Cancer development	*Veillonella* activates PI3K signaling pathway to participate in tumor development.	[68]
	Breast Cancer	*Enterotoxigenic Bacteroides fragilis (ETBF)*	Tumor promotion	Proliferation and migration	*ETBF* triggers breast cancer growth and metastasis through β-catenin and Notch1 pathways.	[69]
		*Faecalibacterium prausnitzii*	Tumor suppression	Proliferation	*F. prausnitzii* suppresses the growth of breast cancer cells through inhibition of IL-6/STAT3 pathway.	[70]

*FadA* Fusobacterium adhesin A, *STAT3* Signal transducer and activator of transcription3, *TLR2* toll-like receptor2, *TLR4* toll-like receptor4, *ROS* reactive oxygen species, *PCWBR2* putative cell wall binding repeat 2, *SGMB Streptococcus gallolyticus* member bacteria, *IL-1* interleukin 1, *COX-2* cyclooxygenase-2, *IL-8* interleukin 8, *CCR5* CC chemokine receptor 5, *VCAM1* Vascular cell adhesion molecular 1, *CAF* cancer-associated fibroblasts, *ESCC* esophageal squamous cell carcinoma, *IL-1β* interleukin 1β, *IL-23* interleukin 23, *PI3K* phosphatidylinositol-3 kinase, *HCC* hepatocellular carcinoma, *PDCD4* programmed cell death 4, *AP-1* activating protein-1

### Autophagy and cancer

**Fig. 2:**
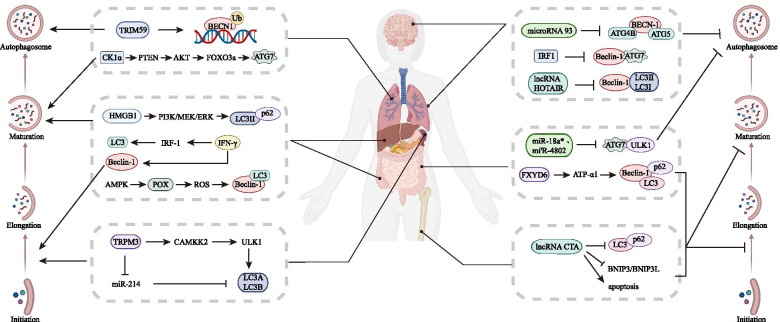
Different proteins or small molecules regulate autophagy pathways. Autophagy is a key regulator during tumorigenesis. It can not only promote cancer by providing nutrition for tumor cells but can also inhibit cancer progression by increasing apoptosis. Autophagy can be divided into four parts: initiation, elongation, maturation, and the fusion of autophagosomes and lysosomes. ATGs participate in each step and eventually promote or inhibit cancer

Autophagy is a degradation pathway by which eukaryotic cells degrade damaged organelles and proteins through lysosomes and is widely found in both normal cells and malignant tumor cells [71, 72]. The process of autophagy mainly includes the formation and extension of isolation membranes or phagocytic bubbles, the formation of autophagosomes, the fusion of autophagosomes and lysosomes to form autophagolysosomes, and the final degradation of intracellular substances [17]. Degradation products, such as amino acids and fatty acids, can be reused by cells [4], which is considered to be a favorable repair and defense mechanism [73]. Gozuacik and other scholars have pointed out that the occurrence of some malignant tumors is accompanied by the inhibition of autophagy [74]. A large number of studies have found that autophagy is closely but complexly related to malignant tumors, particularly affecting the processes of recurrence, metastasis and drug resistance [75] (Fig. 2). TRPM3 is highly expressed in clear cell renal cell carcinoma and induces a high level of autophagy by activating the upstream CAMKK2/ULK1 cascade and inhibiting endogenous miR-214 through the CAMKK2/AMPK pathway, ultimately promoting tumor growth [76]. TRIM59, which contains tripartite motifs, inhibits the NF-κB pathway, downregulates the transcription of BECN1, and affects the ubiquitination level of BECN1 via TRAF6-induced K63 linkage at the same time. As a result, it blocks the establishment of the BECN1/PIK3C3 complex, which triggers the downstream autophagy cascade [77]. POX induces protective autophagy, which can promote HT-29 cell survival in the hypoxic tumor microenvironment through AMPK activation [78]. In non-small-cell lung cancer (NSCLC), casein kinase 1 alpha (CK1α), an autophagy inducer, activates the PTEN/AKT/FOXO3a/ATG7 axis, which increases autophagy and suppresses tumor progression to negatively regulate tumor growth [79]. IFN-γ induces the formation of autophagosomes and the conversion of LC3 through the IRF-1 signaling pathway, which aids to construct the autophagy complex. IFN-γ not only inhibits cell growth, rather it induces non-apoptotic cell death in Huh7 HCC cells [80]. In addition, IFN-γ can also upregulate the expression of Beclin-1, which is vital in autophagy in gastric epithelial cells, and can suppress IL-1β-induced inflammation, *H. pylori*-induced epithelial apoptosis, cell proliferation, and Dckl1^+^ cell elevation to aid in inhibiting bacterial infection and gastric mucosa carcinogenesis [81]. Knockdown of lncRNA HOTAIR with siRNA reduces autophagy, inhibits EMT, decreases cell viability, suppresses cell proliferation, induces cell apoptosis, and enhances sensitivity to radiotherapy in radioresistant HeLa cells through downregulating the activity of the Wnt/β-catenin signaling pathway [82]. When overexpressed, lncRNA CTA inhibits autophagy by decreasing the level of LC3-II isoforms and BNIP3/BNIP3L expression. It also promotes cell apoptosis induced by chemotherapy in osteosarcoma [83]. MiR-93 can suppress autophagic activity by downregulating the expression of BECN1, ATG4B, ATG5, and SQSTM1 in glioblastoma stem cells (GSCs), which results in the suppression of tumor cell growth and glioma sphere self-renewal and the enhancement of temozolomide (TMZ) activity to combat tumor progression by inhibiting autophagy [84]. In CRC, miR-18a* and miR-4802, which target ULK1/ATG7, regulate autophagy via the TLR4 and MYD88 signaling pathways. Both of these miRNAs promote CRC chemoresistance to oxaliplatin and 5-FU [36]. The silencing of FXYD6 promotes prosurvival autophagy and inhibits apoptosis by regulating the activity of ATP-α1, whereas the overexpression of FXYD6 increases chemosensitivity in CRC [85]. Knockdown of IRF1, which negatively correlates with ATG7, increases the level of autophagy by blocking IGF1 receptor and BECN1 expansion, thus promoting resistance to antiestrogens in breast cancer [86]. HMGB1 induces autophagy by increasing LC3-II expression, decreasing p62 expression and inhibiting the formation of autophagosomes through the PI3K/MEK/ERK pathway, thus promoting chemotherapy resistance in leukemia cells [87]. Studies have suggested that the absence of autophagy might lead to cancer development, but autophagy itself may also promote tumorigenesis [88, 89]. To a large extent, changes in autophagy levels may increase autophagy induction or inhibit autophagy activity in tumor cells, but in general, there are three main situations in which changes occur: first, the hypoxic “low-nutrient environment” of the tumor leads to increased autophagy [90]; second, some cancer-related genes are closely related to the autophagy process, and their aberrations lead to changes in autophagy activity [91]; third, changes in lysosomal activity and transport capacity in tumor cells may also lead to large variations in autophagy levels [92]. Autophagy levels vary among different types of tumor cells and different stages of tumorigenesis and even in different parts of tumor tissue [93]. Consistent with the complex changes in autophagy activity, the effects of autophagy on cancer also require special analysis. First, autophagy can provide tumor cells with the metabolites needed for growth and maintain the stability of the internal environment, thus promoting cancer [94–96]. Second, autophagy can avoid the threats of oxidative stress, persistent inflammation, and DNA damage and thus playing a role in inhibiting cancer [97]. The above molecules and their mechanisms of regulating autophagy in tumor progression are summarized in Table 2.

**Table 2:** Regulatory factors regulating autophagy and tumor behaviors

Regulatory factors	ATGs	Stage of autophagy	Cancer type	Tumor promotion /Tumor suppression	Tumor Behavior	Molecular mechanism	Ref.
TRPM3	ULK1 LC3	Induce autophagy initiation and maturation	Clear cell renal cell carcinoma	Tumor promotion	Proliferation	TRPM3 promotes autophagy through miR-214 and CAMKK2-ULK1 cascade, thus supports the ccRCC cell growth.	[76]
TRIM59	Beclin-1	induce autophagy nucleation	Non-small cell lung cancer	Tumor promotion	Tumor progression	TRIM59 promotes the transcription and the ubiquitination of BECN1 to facilitate tumor progression.	[77]
POX	Beclin-1 LC3	Induce autophagy nucleation and maturation	Colorectal cancer	Tumor promotion	Tumor survival	POX induces autophagy activation and promotes tumor cell survival in hypoxic TME.	[78]
CK1α	ATG7	Induce autophagy maturation	Lung tumor	Tumor suppression	Proliferation	CK1α activates PTEN/AKT/FOXO3a/ ATG7 axis to induce autophagy and suppress lung tumor growth.	[79]
IFN-γ	LC3	Induce autophagy maturation	Hepatocellular carcinoma	Tumor suppression	Proliferation	IFN-γ induces autophagy through IRF-1 to inhibit tumor growth.	[80]
IFN-γ	Beclin-1	Induce autophagy nucleation	Gastric cancer	Tumor suppression	Tumorigenesis	IFN-γ induces autophagy through upregulation of Beclin-1 for inhibiting tumorigenesis.	[81]
HOTAIR	Beclin-1 LC3	Inhibit autophagic activity	Cervical cancer	Tumor promotion	EMT process and radioresistance	HOTAIR attenuates sensitivity to radiotherapy by reduction of autophagy and reversal of EMT via Wnt signaling.	[82]
CTA	LC3 p62	Inhibit autophagy maturation	Osteosarcoma	Tumor suppression	Apoptosis and chemoresistance	CTA promotes apoptosis and reduces chemoresistance via downregulating BNIP3/BNIP3L and autophagy.	[83]
miR-93	Beclin-1 ATG4B ATG5	Inhibit autophagy nucleation and maturation	Glioblastoma	Tumor promotion	Chemoresistance	miRNA-93 inhibits multiple autophagy protein and reduces chemoresistance in GSCs.	[84]
miR-18a* miR-4802	ATG7 ULK1	Inhibit autophagy initiation and maturation	Colorectal cancer	Tumor promotion	Chemoresistance	Selective loss of miR-18a*/4802 activates cancer autophagy and enhances CRC chemoresistance.	[36]
FXYD6	Beclin-1 LC3 p62	Inhibit autophagy nucleation and maturation	Colorectal cancer	Tumor suppression	Chemoresistance	FXYD6 regulates cell autophagy via ATP-α1 activity and decreases chemotherapy resistance.	[85]
IRF1	Beclin-1 ATG7	Inhibit autophagy nucleation and maturation	Breast cancer	Tumor suppression	Chemoresistance	IRF1 inhibits the formation of autophagic vacuole and BECN1 expression to restore drug sensitivity to ICI.	[86]
HMGB1	LC3 p62	Induce autophagy maturation	Leukemia	Tumor promotion	Chemoresistance	HMGB1 induces autophagy through the PI3K/MEK/ERK pathway, thus promotes chemotherapy resistance.	[87]

*TRPM3* transient receptor potential melastatin-3, *ULK1* unc-51-like kinase 1, *LC3A* light chain 3A, *LC3B* light chain 3B, *CAMKK2* calcium-calmodulin-dependent protein kinase kinase-2, *TRIM59* tripartite motif 59, *BECN1* Beclin1, *POX* Proline Oxidase, *CK1α* casein kinase 1 α, *ATG7* autophagy related 7, *IFN-γ* interferon γ, *IRF1* interferon regulatory factor 1, *EMT* epithelial-mesenchymal transition, *DOX* doxorubicin, *BNIP3* Bcl-2-interacting protein 3, *BNIP3L* BCL-2-interacting protein 3 like, *ATG4B* autophagy related 4B, *ATG5* autophagy related 5, *GSC* glioblastoma cell, *FXYD6* FXYD domain containing ion transport regulator 6, *ICI* immune checkpoint inhibitors, *HMGB1* high mobility group box-1

## Crosstalk between microbiota and autophagy in cancer development

**Fig. 3:**
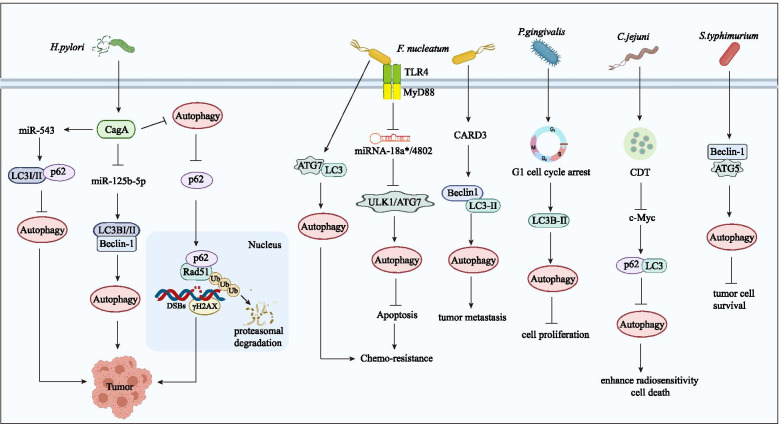
Crosstalk between autophagy and microbiota in cancer. The crosstalk between autophagy and microbiota regulates multiple physiological and pathological responses, including cancer progression. Autophagy can play a role in microbiota-mediated tumorigenesis, metastasis and drug resistance in different cancers. *H. pylori* regulates miR-543, miR-125b-5p and autophagy through its virulence factor CagA to promote tumor occurrence. *F. nucleatum* mainly regulates autophagy through TLR/MyD88 and its downstream miRNA-18a^*^/4802 and then plays a cancer-promoting role in colorectal cancer. *P. gingivalis* can induce G1 cell cycle arrest. *C. jejuni* can enhance cell radiosensitivity by producing cytolethal distending toxin (CDT). *S. typhimurium* inhibits tumor cell survival through autophagy

*H. pylori* is a major risk factor for gastric cancer [98]. Studies have shown that approximately 2% ~ 3% of people infected with *H. pylori* eventually develop gastric cancer [99]. Studies have also shown that autophagy can be induced by the concerted action of *H. pylori* and virulence factors in tumor cells [100]. Cytotoxic-related gene A (CagA) and vacuolar toxin A (VacA) are the main pathogenic factors of *H. pylori*, and both factors are related to autophagy and gastric cancer [101] (Fig. 3). CagA was reported to inhibit autophagy by activating the PI3K/AKT/mTOR pathway [102]. As soon as CagA enters gastric epithelial cells, it may be phosphorylated by tyrosine, which is activated by many signaling factors, therefore changing the cytoskeleton of polyactin, inducing an inflammatory response, initiating apoptosis, suppressing autophagy, and leading to cell scattering [103–107]. VacA is another main factor mediating the involvement of *H. pylori* in regulating autophagy [108]. When gastric mucosal cells are exposed to VacA for a short period of time, autophagy levels can increase, therefore inhibiting tumor growth [103]. When gastric epithelial cells are exposed to VacA for a long period of time, antiphagocytic pathways are affected [109]. Long-term *H. pylori* infection decreases the level of autophagy and induces the collection of the autophagic substrate p62, which can subsequently interact with Rad51, a DNA repair marker, directly through its UBA domain, resulting in the promotion of Rad51 ubiquitination and degradation, thus suppressing the capability to repair damaged DNA. In addition, *H. pylori* might promote gastric tumorigenesis by promoting double-strand breaks (DSBs) and genomic instability [110]. Therefore, eradicating *H. pylori* and increasing autophagy can help prevent gastric cancer [111, 112]. At present, the mechanism of autophagy in promoting gastric cancer formation by *H. pylori* is unclear, thus further studies are mandated.

As mentioned above, *F. nucleatum* is closely related to the occurrence and development of CRC [50]. With the development of CRC, the abundance of *F. nucleatum* in tumor tissues increases gradually compared to that in normal tissues [113]; this finding has also been supported by the positive correlation between *F. nucleatum* and the American Joint Committee on Cancer (AJCC) stage of CRC patients [114]. Fang JY from Shanghai Institute of Digestive Diseases pointed out that *F. nucleatum* can promote resistance to chemotherapeutic drugs in CRC patients by affecting autophagy-regulating miRNAs [36]. For other cancers, studies have shown that *F. nucleatum* regulates the expression of endogenous LC3 and ATG7 and the formation of autophagosomes, resulting in chemoresistance to 5-FU, cisplatin (CDDP), and docetaxel [115].

## The role of microbiota in autophagy-related cancer

### *Helicobacter pylori*

The detailed mechanism of the occurrence and development of gastric cancer mediated by *H. pylori* is still unclear and is influenced by many variables, including strain-specific bacterial components, the complex role of the inflammatory response, the diversity of host heredity, environmental impact, and so on. Recent studies have revealed that megakaryocyte autophagy induced by *H. pylori* is a conserved process by which eukaryotic cells maintain intracellular environmental stability and combat external stress, thus promoting tumor progression [116, 117]. It has also been reported that *H. pylori* can escape autophagy by downregulating the expression of autophagy proteins and can facilitate gastric carcinogenesis [118]. The mechanism of autophagy caused by *H. pylori* infection is complex in gastric cancer, and autophagy might have different functions at different infection stages [119]. Terebiznik first reported that *H. pylori* triggered autophagy in gastric adenocarcinoma epithelial cells and identified LC3 as an autophagy-related marker [104]. Furthermore, Yahiro et al. demonstrated that low-density lipoprotein receptor-associated protein-1 (LRP-1) could mediate autophagy through VacA in gastric cancer epithelial cells, which might be associated with the activation of VacA channels to promote the formation of tyrosine phosphatase-related vacuoles [120]. As another important virulence factor of *H. pylori*, CagA has been suggested to trigger the overexpression of miR-543 (Fig. 3), which can suppress autophagy by interacting with SIRT1, to increase EMT and promote tumor cell migration and invasion in gastric cancer [121].

In recent years, a growing number of scholars have reported that the occurrence of colon cancer is closely related to *H. pylori* infection [122]. Pyrogallol treatment significantly reduced the viability rate of *H. pylori* to 62% and exhibited marked antimetastatic potential via inhibiting the migration of HT-29 cells [123]. IHC staining was applied and demonstrated that Beclin-1 was abnormally expressed in colon cancer tissue infected with *H. pylori.* Therefore, it was speculated that Beclin-1 mediated the tumor-promoting activity of *H. pylori*. Moreover, CagA-positive *H. pylori* might decrease the level of miR-125b-5p (Fig. 3), resulting in high expression of LC3-II/LC3-I and Beclin-1, which are key molecules related to autophagy in colon cancer, thereby inducing the autophagy, proliferation and invasion of colon cancer cells, hence inducing colon cancer [124]. In addition to regulating the autophagy and apoptosis of colon cancer cells, *H. pylori* can inhibit gastric acid secretion, indirectly causing the excessive release of gastrin, which leads to the abnormal proliferation of intestinal cells, inducing colon polyps and even colon cancer [125].

### *Fusobacterium nucleatum*

Available data show that *F. nucleatum* infection is mainly related to the development of esophageal and pancreatic cancers [41, 48]. In addition, Gallimidi et al. found that *F. nucleatum* plays an essential role in the development of oral cancer. *F. nucleatum* can stimulate the level of IL-6 through the TLR signaling pathway in tumor cells and subsequently activate the STAT3 pathway, thereby promoting the growth of oral squamous cell carcinoma (OSCC) [126]. Fewer studies have investigated the relationship between IL-6 and autophagy in the development of oral cancer. Existing studies revealed that IL-6 can upregulate Beclin-1 and induce autophagy by increasing the level of NS5ATP9 through NF-κB activation; in turn, NS5ATP9 can upregulate IL-6 levels, which subsequently further induced autophagy in liver cancer [127]. Moreover, IL-6 promotes the formation of autophagosomes, strengthens autophagic flux through the IL-6/JAK2/BECN1 pathway and induces chemotherapy resistance in CRC [128]. LC3-II is a key molecule in tumor proliferation and progression, and its expression level can directly reflect the activity of autophagy [129]. Excessive secretion of IL-6 is an adaptive mechanism of autophagy that occurs by means of LC3-II induction; however, there is still no consensus on its effects.

As mentioned above, autophagy plays a critical role in the induction of CRC via *F. nucleatum* infection [48, 50]. *F. nucleatum* can resist chemotherapeutic drugs by regulating autophagy. In vivo and in vitro studies demonstrated that autophagy levels increased in intestinal cancer cells infected with *F. nucleatum*. Western blotting results demonstrated that *F. nucleatum* infection increases the expression of various autophagy signaling elements, such as pAMPK, ATG7, pULK1, and ULK1, in HCT116 and HT-29 cells [36]. The expression levels of miR-18a* and miR-4802 are decreased in intestinal cancer cells infected with *F. nucleatum* to induce chemoresistance, while the expression levels of target genes ULK1 and ATG7 are significantly increased in these cells. Overexpression of miR-18a* and miR-4802 inhibited autophagy induced by *F. nucleatum* in intestinal cancer cells, while inhibition of miR-18a* and miR-4802 had the opposite effect. Therefore, in summary, *F. nucleatum* plays a vital role in inducing CRC chemoresistance through selectively silencing miR-18a* and miR-4802 and activating autophagy pathways [36]. In addition, the *F. nucleatum* concentration was found to be positively related to the level of CARD3, which was lower in those without metastasis. Downregulation of CARD3 decreased the expression of proteins related to migration, invasion, metastasis, and autophagy and the formation of autophagosomes induced by *F. nucleatum* in vitro or in vivo. Thus, *F. nucleatum* was also demonstrated to activate autophagy by targeting CARD3 to promote CRC metastasis [130].

### *Porphyromonas gingivalis*

*P. gingivalis* is an important pathogen causing periodontitis, OSCC, and even esophageal cancer [43, 131, 132]. Bélanger et al. found that the survival of *P. gingivalis* depended upon autophagy activation of endothelial host cells [133]. After *P. gingivalis* invades the cells, the endogenous transport and autophagy process is aberrantly regulated; thus, it can escape degradation by host cells. At the same time, a microenvironment suitable for bacterial colonization and proliferation is created in modified autophagosomes. In addition, *P. gingivalis* infection is considered to promote autophagy and inhibit tumor cell proliferation by inducing G1 cell cycle arrest in host oral cancer cells [35]. The detailed autophagy-related mechanism by which microbiota regulate human cancer is summarized in Table 3.

**Table 3:** Bacteria involved in autophagy-regulated tumors

Bacteria	Autophagy promotion / inhibition	ATGs	Stage of autophagy	Cancer type	Tumor promotion / suppression	Tumor behavior	Molecular mechanism	Ref.
*Helicobacter pylori*	Inhibit autophagy	LC3 p62	Autophagy maturation	Gastric cancer	Tumor promotion	Tumorigenesis	*H. pylori* inhibits autophagy and induces the collection of p62 to promote gastric tumorigenesis.	[110]
	Inhibit autophagy	LC3 p62	Autophagy maturation	Gastric cancer	Tumor promotion	Migration and invasion	CagA promotes miR-543 overexpression which can suppress autophagy, leading to tumor cell migration and invasion.	[121]
	Promote autophagy	LC3 Beclin-1	Autophagy nucleation and maturation,	Colon cancer	Tumor promotion	Proliferation and invasion	CagA-positive *H. pylori* decreases miR-125b-5p level and induce autophagy for promotes the proliferation and invasion of colon cancer.	[124]
*Fusobacterium nucleatum*	Promote autophagy	ULK1 ATG7	Autophagy initiation and maturation	Colorectal cancer	Tumor promotion	Chemoresistance	*F. nucleatum* medicates CRC chemoresistance via deletion of miR18a*/4802 targeting ULK1/ATG7.	[36]
	Promote autophagy	ATG7 LC3	Autophagy maturation	Esophageal squamous cell carcinoma	Tumor promotion	Chemoresistance	*F. nucleatum* induces ESCS chemoresistance by modulating the endogenous LC3 and ATG7 expression, as well as autophagosomes formation.	[115]
	Promote autophagy	LC3 Beclin1	Autophagy nucleation and maturation,	Colorectal cancer	Tumor promotion	Metastasis	*F. nucleatum* promotes CRC metastasis by activating autophagy signaling via CARD3.	[130]
*Porphyromonas gingivalis*	Promote autophagy	LC3	Autophagy maturation	Oral cancer	Tumor suppression	Proliferation	*P. gingivalis* infection promotes autophagy and controls cell proliferation and G1 arrest in host oral cancer cells.	[35]
*Salmonella typhimurium*	Promote autophagy	ATG-5 Beclin1	Autophagy maturation	Liver cancer Gastric cancer	Tumor suppression	Proliferation	Inhibiting autophagy enhances the cancer-cell killing ability of *S. typhimurium*.	[134]
*Campylobacter jejuni*	Inhibit autophagy	ATG-5 ATG-12	Autophagy maturation	Prostate cancer	Tumor suppression	Radioresistance	*C. jejuni* suppresses autophagy via CDT and then enhances PC cells radiosensitivity.	[135]

*LC3* light chain 3, *CagA* cytotoxin-associated gene A, *ULK1* unc-51-like kinase 1, *ATG7* autophagy-related gene 7, *CARD3* caspase activation and recruitment domain 3, *CDT* cytolethal distending toxin

## Targeting microbiota/autophagy in cancer therapy

Since autophagy plays an important role in the occurrence and development of tumors, it may promote tumor formation, or inhibit tumor cell growth and metastasis through certain mechanisms. In recent years, breakthrough results have been obtained in clinical trials assessing the relationship between autophagy and cancer. A series of drugs targeting autophagy have also been introduced through animal experiments or clinical trials. In the following section, we systematically summarized the drugs that individually target autophagy and exert anticancer effects and their mechanisms.

Alternatively, the abovementioned microbial community members, especially *H. pylori,* may indirectly cause cancer by promoting mucositis or causing systemic diseases. Studies have shown that *H. pylori* is one of the most important risk factors for gastric cancer. Therefore, we considered whether inhibiting the carcinogenic microbial community in the tumor microenvironment can delay the occurrence of tumors and even play an anticancer role. Similarly, we also summarized drugs targeting the microbial community in the tumor microenvironment and their mechanisms, as well as the corresponding clinical trials.

Most importantly, we emphasized the critical role of autophagy in mediating microbial communities and cancer. A variety of bacteria regulate tumor formation, development, invasion and metastasis by inducing or inhibiting autophagy. Therefore, the simultaneous use of drugs targeting autophagy and microorganisms may play a synergistic anticancer role. However, few such studies have been performed, and more clinical trials are urgently needed to better understand the therapeutic potential of drugs targeting the microbiota/autophagy axis, which could help to develop new drugs to prevent or treat human cancer.

### Clinical trials of drugs targeting autophagy

Chloroquine (CQ) is a quinoline derivative belonging to the heterocyclic aromatic compound family and has been used as an antimalarial therapy for many years [145]. Moreover, CQ and its derivatives block autophagy by regulating lysosomal function [146]. However, there are few animal experiments or clinical trials on its anticancer activity as a monotherapy drug. Studies have shown that CQ can inhibit autophagy and enhance the efficacy of many anticancer agents in a variety of mouse cancer models, such as colon cancer [147, 148], head and neck cancer [149] and gallbladder cancer [150]. We found clinical trials that attempted to investigate the antitumor activity of CQ in small cell lung cancer (SCLC) and breast cancer [151] patients, as shown in Table 4.

**Table 4:** Autophagy related drugs and associated clinical trials

Drugs	Cancer type	Clinical trial / Animal experiment	Phase	Ref. / Trial ID
Chloroquine	Small cell lung cancer	Clinical trial	Phase I	NCT00969306
	Small cell lung cancer	Clinical trial	Phase I	NCT01575782
	Breast cancer	Clinical trial	Phase II	NCT02333890
Hydroxychloroquine	Estrogen receptor-positive breast cancer	Clinical trial	Phase Ib/II	NCT02414776
	Prostate carcinoma	Clinical trial	Phase I	NCT02421575
	Solid tumors	Clinical trial	Phase I	NCT03015324
	Melanoma	Clinical trial	Phase I	NCT00962845
	Hepatocellular carcinoma	Clinical trial	Phase I/II	NCT02013778
	B-CLL	Clinical trial	Phase II	NCT00771056
	Renal cancer	Clinical trial	Phase Ib	NCT01144169
Sorafenib	Hepatocellular carcinoma	Clinical trial	Phase Ib/II	NCT03211416
Lys05	Melanoma, colon cancer	Animal experiment	/	[136]
DQ661	Melanoma, pancreatic cancer, colorectal cancer	Animal experiment	/	[137, 138]
SAR405, SB02024	Melanoma, colorectal cancer	Animal experiment	/	[139]
Mefloquine	Pancreatic ductal adenocarcinoma	Animal experiment	/	[140]
Spautin-1	Prostate cancer	Animal experiment	/	[141]
	Melanoma	Animal experiment	/	[142]
Rapamycin	Bladder cancer	Clinical trial	Phase II	NCT04375813
	Advanced cancers	Clinical trial	Phase Ib	NCT00707135
	HNSCC	Animal experiment	/	[143]
	Lung squamous cell carcinoma	Animal experiment	/	[144]
Temsirolimus	Prostate cancer	Clinical trial	Phase II	NCT00919035
	Advanced cancers	Clinical trial	Phase I/II	NCT00877773
	HNSCC	Clinical trial	Phase II	NCT01172769
	Advanced bladder cancer	Clinical trial	Phase II	NCT01827943
	Cervical cancer	Clinical trial	Phase II	NCT01026792
	Metastatic neuroendocrine carcinoma	Clinical trial	Phase II	NCT00093782
	Liver cancer	Clinical trial	Phase II	NCT01079767
	Endometrial carcinoma	Clinical trial	Phase IIa	NCT02093598

*HNSCC* head and neck squamous cell carcinoma, Trial ID registered number at Clinical Trials.gov

Hydroxychloroquine (HCQ) is a derivative of CQ that has an extra hydroxyl group; however, it has 40% lower toxicity than that of CQ. Moreover, HCQ has stronger anti-inflammatory effects; therefore, it has been used to treat rheumatoid arthritis and systemic lupus erythematosus [152, 153]. Similarly, HCQ can increase the cytotoxicity of various chemotherapies and target therapies by inhibiting autophagy [154]. However, a phase II clinical trial and pharmacodynamic study of HCQ in metastatic pancreatic adenocarcinoma patients showed that HCQ alone could induce severe autophagy inhibition but with negligible therapeutic efficacy [155]. A clinical trial (NCT02013778) was developed to confirm the dose limiting toxicity and maximum tolerated dose of the oral administration of HCQ combined with transarterial chemoembolization (TACE) in the management of hepatocellular carcinoma (HCC). Furthermore, another phase 0 clinical trial, NCT00962845, investigated the role of HCQ in disrupting autophagy, which may promote the survival of cancer cells under chemotherapy in prostate cancer (PCa). Other clinical studies on HCQ are summarized in Table 4**.** In addition, there are other micromolecular drugs that can play anticancer roles by regulating autophagy. Compared with HCQ, Lys05, a water-soluble salt of Lys01 that has a 10-fold greater ability to inhibit autophagy than HCQ and can improve the degradation of lysosomes more potently, ultimately downregulating autophagy and tumor growth. Contrary to HCQ, Lys05 shows single-agent antitumor activity and less toxicity at lower doses in animal models [136]. By adjusting the structure of Lys05, DQ661 emerged and has been regarded as an anticancer compound that inhibits mTOR and autophagy. DQ661, as the most important lysosomal inhibitor [137], can promote DNA damage independent of apoptosis, decrease autophagic flux and contribute to lysosomal membrane permeability (LMP). DQ661 treatment significantly reduced the tumor volume compared with that of the control mice, and the weight of the mice did not significantly change. Immunoblotting revealed that mTORC1 and autophagy were inhibited in a melanoma mouse model. In addition, DQ661 improved survival in a colon cancer mouse model and showed antitumor activity in a syngeneic pancreatic model that was resistant to immunotherapy [138]. SAR405, a selective PIK3C3/Vps34 inhibitor, can prevent the formation of late endosome and lysosome compartments by inhibiting the activity of PIK3C3 kinase and can suppress autophagy and mTOR signaling synergistically in tumor cells. Vps34i inhibits autophagic flux in multiple cancer mouse models, including melanoma, CRC and renal cell carcinoma models. Systemic treatment (oral gavage) with Vps34i contributed to significant tumor weight reduction, tumor growth suppression and survival improvement in cancer-bearing mouse, which means that SB02024 and SAR405 (Vps34i) do not target one specific cancer type for combating cancer, and both might be used in multiple tumor models [139]. 3-Methyladenine (3-MA) [156], wortmannin [157] and LY294002 [158] are all PI3K inhibitors that inhibit autophagy by disrupting the production of PI3P through inhibiting the class III PI3K complex [159], which plays a vital role in recruiting other ATG proteins for the activation of autophagy at the isolation membrane [160, 161]. The effects of 3-MA and wortmannin in regulating autophagy are slightly different. 3-MA can promote autophagy in complete medium when applied for a long time, but it can also inhibit starvation-induced autophagy, as shown in previous studies. Nevertheless, wortmannin is capable of suppressing autophagy in any nutrient status. 3-MA promotes autophagy by transiently suppressing class III PI3K, while wortmannin persistently inhibits class III PI3K [162]. Many clinical trials have been developed to explore the anticancer effects of 3-MA and wortmannin in combination with other cancer treatments. For example, 3-MA inhibits autophagy, which might be a resistance mechanism of colon cancer cells against 5-FU, and enhances the apoptosis of colon cancer cells treated with 5-FU; thus, inhibition of autophagy could decrease the chemoresistance of colon cancer [163]. Lin J et al. demonstrated that wortmannin could markedly enhance the antitumor effect of Ag nanoparticles (NPs) in a B16 mouse melanoma cell model [164]. However, there are few clinical trials on the single-agent antitumor activity of 3-MA and wortmannin; therefore, more work needs to be done on this topic. Mefloquine hydrochloride suppresses the expression of lysosomal LAMP1/LAMP2, which plays an important role in the formation of autolysosomes and inhibits CD133/CD44v9 colon cancer stem cells (CSCs) to exert its antitumor effect. Notably, mefloquine alone can achieve almost the same effects as other anticancer agents [165]. Spautin-1 was developed for autophagy attenuation and can induce the degradation of PI3K/Vps34 complexes by inhibiting USP10 and USP13, two ubiquitin-specific peptidases targeting the Beclin-1 subunit [141]. Additionally, spautin-1 has also been shown to suppress the phosphorylation of EGFR and the activation of its downstream signaling, contributing to cell cycle arrest and programmed cell death in PCa in a USP10/USP13 independent manner [166]. In melanoma, spautin-1 can decrease tumor growth and enhance the antitumor effect of cisplatin by targeting USP10 and USP13 [142]. Rapamycin (sirolimus), a macrolide immunosuppressant, was first isolated from *Streptomyces hygroscopicus* and was found to inhibit mTOR protein kinase, which plays an important role in downregulating autophagy [143, 167]. Temsirolimus is an ester of rapamycin that can selectively inhibit mTOR kinase and subsequently disrupt cell cycle regulatory protein translation and then exert an anticancer effect [168]. Both rapamycin and temsirolimus can modulate autophagy by inhibiting the mTOR pathway, and some associated clinical trials have started to study their anticancer effects in various cancer entities. Autophagy-related drugs and related clinical trials are summarized in Table 4**.**

### Clinical trials of drugs targeting microbiota

*H. pylori* infection is an important risk factor for gastrointestinal diseases, such as gastric inflammation, gastric cancer and gastric mucosa-related lymphoid-tissue lymphoma [169]. Therefore, the eradication of *H. pylori* may inhibit the occurrence of gastric cancer. Traditionally, the standard scheme for the eradication of *H. pylori* is the triple therapy of anti-secretory agents, such as proton pump inhibitors, combined with two kinds of antibiotics, such as amoxicillin, levofloxacin, clarithromycin, and metronidazole [169]. In addition, phytomedicines and probiotics have also been used for the treatment of *H. pylori* infection in recent studies [170]. The purpose of NCT04660123 is to observe the eradication rate of *H. pylori* infection, the improvement of symptoms, and the incidence of adverse effects in gastric cancer patients treated with bismuth colloidal pectin granule quadruple therapy. Itraconazole is a broad-spectrum antibiotic and is a triazole antifungal agent, which has commendable pharmacodynamic and pharmacokinetic profiles and is broadly applied for preventing or treating systemic fungal infections [171, 172]. Recent studies demonstrated that itraconazole can induce autophagy, downregulate the expression of steroid carrier protein 2; and redistribute intracellular cholesterol to inhibit glioblastoma growth [173]. In addition, itraconazole can exert its anticancer effect through the Hedgehog signaling pathway [174]. Itraconazole promotes apoptosis and autophagy by inhibiting the Hedgehog pathway, resulting in a reduction in tumor cells in vitro, and this phenomenon was also observed in a human xenograft breast cancer mouse model, demonstrating its anticancer effect in breast cancer [175]. Itraconazole induces cell cycle arrest and cell apoptosis and then decreases the proliferation, invasion and migration of OSCC cells by inhibiting the Hedgehog pathway. Similarly, itraconazole disrupted the growth of tumor cells, decreased the expression of Ki67, and induced cell apoptosis in the OSCC patient-derived xenograft (PDX) model [176]. Itraconazole can decrease the proliferation and growth of colon cancer cells by promoting autophagy and apoptosis; at the same time, it can reduce the protein expression levels of shh and Gli1 in a dose-dependent manner [177]. Furthermore, the purpose of NCT02749513 is to demonstrate that orally administered itraconazole can inhibit the Hedgehog pathway in patients with esophageal cancer, including adenocarcinoma and squamous cell carcinoma. Probiotics can regulate the gut microbiota, positively affect the interaction between the immune system and microbiota and are beneficial for preventing inflammation and CRC [178]. The purpose of NCT03072641 is to confirm whether probiotic bacteria have a beneficial effect on the colon cancer-associated microbiota and epigenetic alterations. The clinical trials of the above drugs are summarized in Table 5. Taking *Bifidobacterium* orally alone enhances the local control rate of tumors to the same degree as PD-L1-specific antibody therapy, and combination therapy almost stops tumor growth. *Bifidobacterium*-treated mice showed significantly enhanced local tumor control compared with the untreated group, and this effect was accompanied by the accumulation of antigen-specific CD8^+^ T cells in the tumor microenvironment [179]. In addition, oral administration of the probiotic candidate DTA81 might have beneficial effects for preventing CRC development [180]. The relevant clinical trials of the above drugs are shown in Table 5.

**Table 5:** Microbiota related drugs and associated clinical trials

Drugs	Cancer type	Clinical trial / Animal experiment	Phase	Ref. / Trial ID
Bismuth colloidal pectin granules quadruple therapy	Gastric cancer	Clinical trial	Phase IV	NCT04660123
Itraconazole	Breast cancer	Animal experiment	/	[175]
	Oral squamous cell carcinoma	Animal experiment	/	[176]
	Colon cancer	Animal experiment	/	[177]
	Esophageal cancer	Clinical trial	Phase I	NCT02749513
Probiotics	Colon cancer	Clinical trial	Not Applicable	NCT03072641
	Colorectal cancer	Clinical trial	Phase II	NCT00936572
Bifidobacterium	Melanoma	Animal experiment	/	[179]
*Lactobacillus Johnsonii*	Colon Cancer	Clinical trial	Phase II	NCT00936572
*Lactobacillus paracasei* DTA81	Colorectal cancer	Animal experiment	/	[180]

Trial ID registered number at Clinical Trials.gov

### Antitumor effects of antibiotics combined with autophagy inhibitors

At present, there are few antitumor studies evaluating the effect of combined therapy with antibiotics and autophagy inhibitors, and rapamycin is the most commonly used antibiotic. Some scholars attempted to use rapamycin combined with the autophagy inhibitor 3-MA to treat human lung cancer in vitro. Western blotting and MTT results showed that the combination with an autophagy inhibitor could significantly increase the decline in cell survival caused by rapamycin. Liu et al. found that rapamycin and CQ have certain antitumor effects respectively in vitro, and their combined use can enhance the antitumor effect on osteosarcoma [181]. Moreover, rapamycin was further confirmed to promote autophagy by blocking the mTOR pathway in the human osteosarcoma cell line MG63; and CQ was found to enhance apoptosis by blocking autophagy, suggesting that CQ may amplify the effects of rapamycin in inducing apoptosis via autophagy inhibition [182].

## Concluding remarks

Autophagy is a conserved degradation mechanism in eukaryotic cells. The response of eukaryotic cells to external antigens and intracellular aging substances triggers autophagy activation. An imbalance in autophagy is often observed in human cancer. Therefore, it is speculated that autophagy can play a regulatory role in tumor cells, as it can promote tumor growth by providing metabolic energy for tumor cells or inhibit tumor cell proliferation and metastasis by activating certain intracellular signaling pathways. Microbiota are leading causes of cancer that act either by inducing mucosal inflammation or causing systemic disorders. More importantly, bacteria have been globally proven to promote or inhibit cancer via autophagy regulation, suggesting a complex interaction between autophagy and bacteria. This review explains how bacteria regulate tumor development, progression, invasion and metastasis by inducing or inhibiting autophagy and summarizes the influence of various bacteria-mediated autophagy mechanisms on the biological behavior of cancer.

Considering that both autophagy and microbiota can play a certain role in tumor progression and that crosstalk between these factors has been discovered recently, the inhibition of autophagy and/or microbiota with drugs may be beneficial for controlling tumor development. This strategy appears more achievable with the emergence of innovatively developed direct and/or nonspecific small molecule inhibitors of the autophagy/microbiota axis.

## Data Availability

The materials that support the conclusion of this review have been included within the article.
